# Engineered mesenchymal stem cell-derived extracellular vesicles: kill tumors and protect organs

**DOI:** 10.7150/thno.99618

**Published:** 2024-09-23

**Authors:** Yu Li, Yao Wang, Yu Zhang, Yuruchen Zhu, Yuhui Dong, Haobin Cheng, Yinan Zhang, Yue Wang, Zhaoshen Li, Jie Gao

**Affiliations:** 1 Department of Gastroenterology, Changhai Hospital, Naval Medical University, Shanghai, 200433, China.; 2 Stem Cell and Regeneration Medicine Institute, Research Center of Translational Medicine, Naval Medical University, Shanghai, 200433, China.; 3 Changhai Clinical Research Unit, Changhai Hospital, Naval Medical University, Shanghai, 200433, China.; 4 College of Life Science, Mudanjiang Medical University, Heilongjiang Mudanjiang, 157011, China.; 5 Shanghai Key Laboratory of Cell Engineering, Shanghai, 200120, China.; 6 School of Basic Medical Sciences, Naval Medical University, Shanghai, 200433, China.; 7 School of Health Science and Engineering, University of Shanghai for Science and Technology, Shanghai, 200093, China.; 8 School of Chemical Science and Engineering, Tongji University, Shanghai, 200092, China.; 9 Shanghai Institute of Stem Cell Research and Clinical Translation, Shanghai, 200120, China.; 10 National Clinical Research Center for Digestive Diseases, Department of Gastroenterology, Changhai Hospital, Naval Medical University, Shanghai, 200433, China.; 11 National Key Laboratory of lmmunology and Inflammation, Naval Medical University, Shanghai, 200433, China.; 12 Department of Gastroenterology, Changhai Hospital, Naval Medical University, Shanghai, 200433, China.; 13 Shanghai Key Laboratory of Nautical Medicine and Translation of Drugs and Medical Devices, Shanghai, 200433, China.

**Keywords:** solid tumors, mesenchymal stem cell extracellular vesicles, engineering strategies, tissue regeneration, tumor treatment

## Abstract

Solid tumors cause 90% of cancers and remain the primary cause of mortality. However, treating solid tumors presents significant challenges due to the complex tumor microenvironment and drug resistance, leading to inadequate treatment targeting and severe side effects. Surgery, radiotherapy, and chemotherapy Although it is an effective method for the treatment of solid tumors, it can lead to organ dysfunction and affect patient prognosis. Therefore, it is imperative to improve treatment precision and organ repair capabilities to manage solid tumors. Mesenchymal stem cell extracellular vesicles (MSC-EVs) have wide application prospects as a new agent for solid tumor therapy. Firstly, MSC-EVs is a derivative of MSCs. It has the function of promoting tissue regeneration by inducing dedifferentiation in surviving cells after injury. Additionally, MSC-EVs offer unique advantages in terms of safety, stability and penetrability, making them a promising extracellular therapeutic modality for solid tumor treatment. Finally, MSC-EVs are able to enhance therapeutic efficacy through engineering strategies. To sum up, this review takes MSC-EVs as its object. And then we discuss recent advancements and engineering strategies in the use of MSC-EVs for soid tumor suppression. This review aims to inspire researchers to devise a new method for effectively treat solid tumors.

## 1. Introduction

Cancer remains the preeminent cause of mortality globally, posing a formidable challenge to life expectancy enhancements 1. The World Health Organization's 2019 data underscore cancer as a top-two killer before age 70 in 112 nations and a top-four cause in an additional 2,3. This prominence highlights the relative success in reducing mortality from stroke and coronary heart disease, in stark contrast to the persistent threat of cancer 1. The 2021 Global Cancer statistics reveal a disparity: while solid tumors constitute about 90% of all cases, they represent only 40% of clinical trials, predominantly in early phases 4. The efficacious and safe management of solid tumors is hindered by two principal obstacles. Firstly, these tumors are shielded by a fibrous matrix and immunosuppressive cells, which impede immune cell attacks 5. Secondly, the low pH and oxygen levels within the dense core of these tumors impede drug penetration 4. Surgery, a prevalent treatment, can curb tumor progression and metastasis 6,7, yet it is not without its limitations. Post-surgical challenges include the uncertainty of complete cancer eradication, leading to recurrence, and the difficulty of regenerating healthy tissue amidst chronic inflammation and extensive tissue damage 8,9. Post-resection treatment periods thus present the dual challenge of preventing local recurrence and fostering healthy tissue regeneration.

Extracellular vesicles (EVs), enveloped by a lipid bilayer and sized between 40 to 160 nm, have been identified as pivotal in intercellular communication 10,11. They facilitate the transfer of functional biomolecules, enabling cells to exchange signals and information. Mesenchymal stem cells (MSCs) are notable for their secretion of bioactive substances with antiapoptotic, immunomodulatory, and proangiogenic capabilities, which are instrumental in tissue and organ regeneration. MSC-derived EVs (MSC-EVs) mirror these therapeutic properties, offering a similar regenerative potential. Notably, MSC-EVs can be preserved for extended periods while retaining their biological activity and are nonimmunogenic, making them an attractive therapeutic option for post-surgical tissue repair following solid tumor removal. Despite their promise, native MSC-EVs may not sufficiently prevent local tumor recurrence post-resection. Thus, the engineering of MSC-EVs is crucial for enhancing their therapeutic efficacy in solid tumor treatment. Engineered EVs can be designed to deliver specific therapeutic agents to targeted sites with spatial precision, allowing for a sustained release of therapeutic molecules and an extended duration of action. However, the literature on the advancement of engineered MSC-EVs in postoperative solid tumor management remains limited, with only a handful of reviews addressing this topic.

In this review, we intend to delineate the therapeutic potential of MSC-EVs in solid tumor treatment, encompassing both organ regeneration and tumor suppression. We will juxtapose the merits and limitations of MSC-EVs, scrutinizing the nexus between their intrinsic biological attributes and the engineering strategies that could refine their application in oncology. Additionally, we will explore the impediments to the deployment of MSC-EVs, project their prospective applications, and propose innovative tactics and design paradigms to augment their potency in tumor containment and tissue restoration.

## 2. Introduction to MSC-EVs

### 2.1 Biogenesis of MSC-EVs

**Figure 1:**
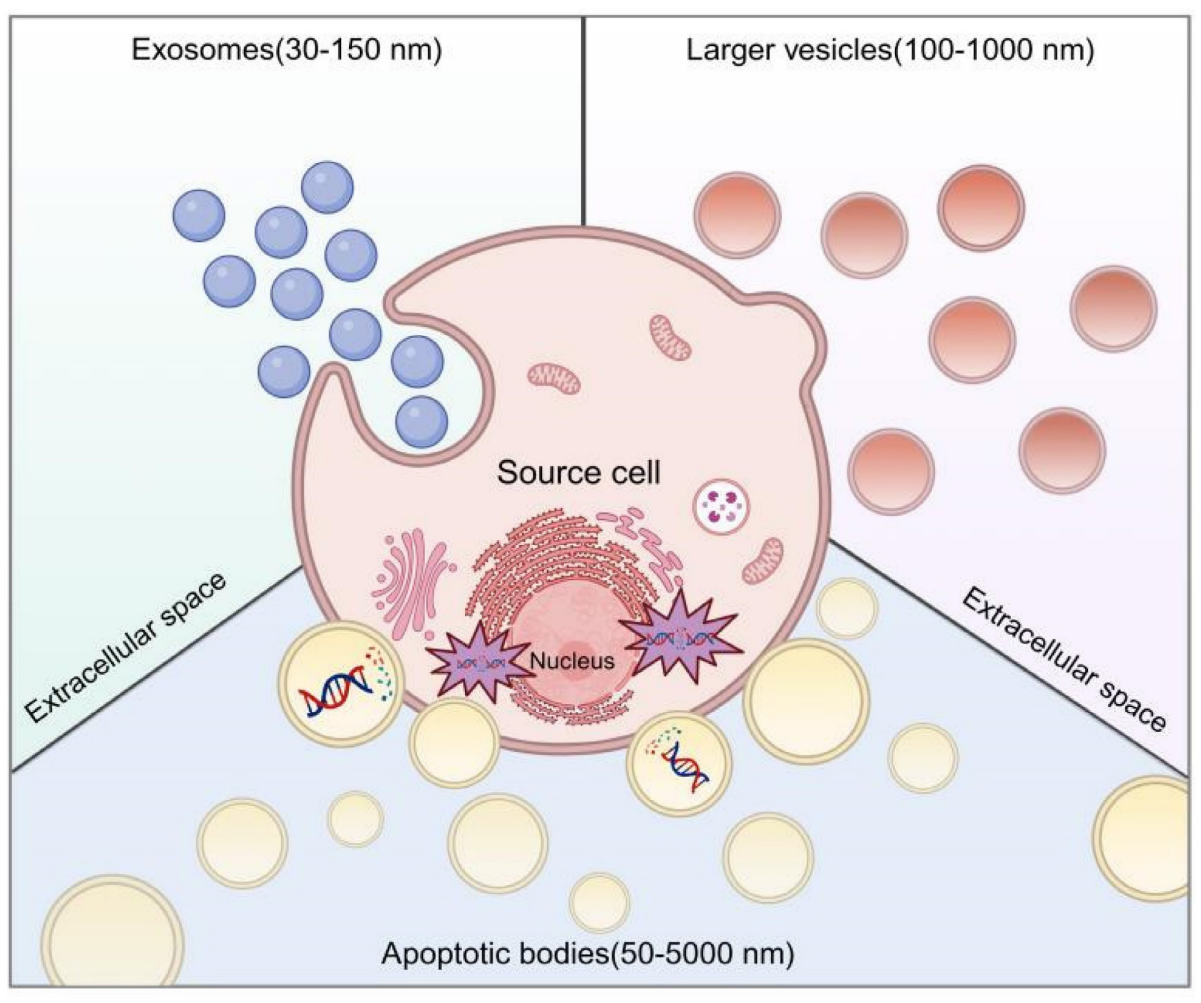
Biogenesis of MSC-EVs. Created using BioRender.com.

We clarify the classification of extracellular vesicles (EVs) based on size, identifying three distinct subtypes. The initial category encompasses large vesicles, termed ectosomes or microvesicles, which emerge through the budding and release from the plasma membrane, exhibiting sizes from 100 to 1000 nm. They directly extrude into the extracellular matrix. The second category consists of exosomes, 30 to 150 nm in diameter, arising from the endosomal membrane's invagination, culminating in the creation of intraluminal vesicles (ILVs) within multivesicular bodies (MVBs) 12. The final category is apoptotic bodies (ApoBDs), secreted into the extracellular space through the blebbing of apoptotic cells, with diameters varying widely from 50 nm to 5000 nm, a discrepancy noted in the literature. The delineation of EV subtypes is often obscured by size overlaps and nomenclature inconsistencies. Accurate identification of EV subtypes without specific markers remains a challenge. Until a more definitive set of criteria is established, EVs will continue to be employed as an umbrella term for these diverse subtypes (**Figure 1**).

### 2.2 Analysis of the content of MSC-EVs

#### 2.2.1 Nucleic acid

Extracellular vesicles (EVs) are minute sacs capable of transporting microRNAs (miRNAs), which are pivotal in the regulation of gene expression. These noncoding RNAs act as key mediators in RNA silencing and the modulation of posttranscriptional gene expression pathways 13. The RNA-laden EVs are instrumental in cellular communication, with a variety of miRNAs, including let-7, miR-1, miR-15, miR-16, miR-181, and miR-375, identified within them, underscoring their role in miRNA regulation 14. Through sequencing technologies, researchers have discerned a multitude of RNA subtypes, indicating the presence of diverse extracellular complexes, such as RNA and RNA-binding proteins (RBPs), within EVs 15-19. The heterogeneous sorting of RNA cargo within EVs suggests a degree of specificity, highlighting the potential regulatory mechanisms of miRNAs in various biological contexts.

#### 2.2.2 Protein

Extracellular vesicles (EVs) are composed of a diverse array of proteins, encompassing those involved in membrane transport and fusion, as well as heat shock proteins (HSPs) such as HSP60, HSP70, and HSP90. They also contain tetraspanins—transmembrane proteins from the four superfamily, including CD9, CD63, CD81, CD82, CD106, and Tspan8—alongside intercellular adhesion molecule (ICAM-1) and proteins linked to multivesicular bodies (MVBs) like ALIX and TSG101. Additional proteins such as integrins, actin, and myosin are also found within EVs 20. Proteomic analysis of mesenchymal stem cell-derived EVs (MSC-EVs) from bone marrow has identified over 700 distinct protein types 21. The protein cargo of EVs is influenced by stress signals, the local microenvironment, and the tissue from which they originate.

#### 2.2.3 Lipids

EVs are distinguished by their unique lipid membrane, which sets them apart from other cellular entities. This membrane is predominantly composed of cholesterol, sphingolipids, and a spectrum of fatty acids, including saturated, monounsaturated, and polyunsaturated varieties 22. It forms the fundamental structure of EVs and is integral to their assembly and the encapsulation of their cargo 23. Unlike the protein and nucleic acid constituents of EVs, the lipid profile does not reveal their tissue of origin. Nonetheless, the lipid composition of EVs is subject to variation, reflecting their diverse contents, biological processes, and functional roles 22.

## 3. Engineering Strategies for MSC-EVs

**Figure 2:**
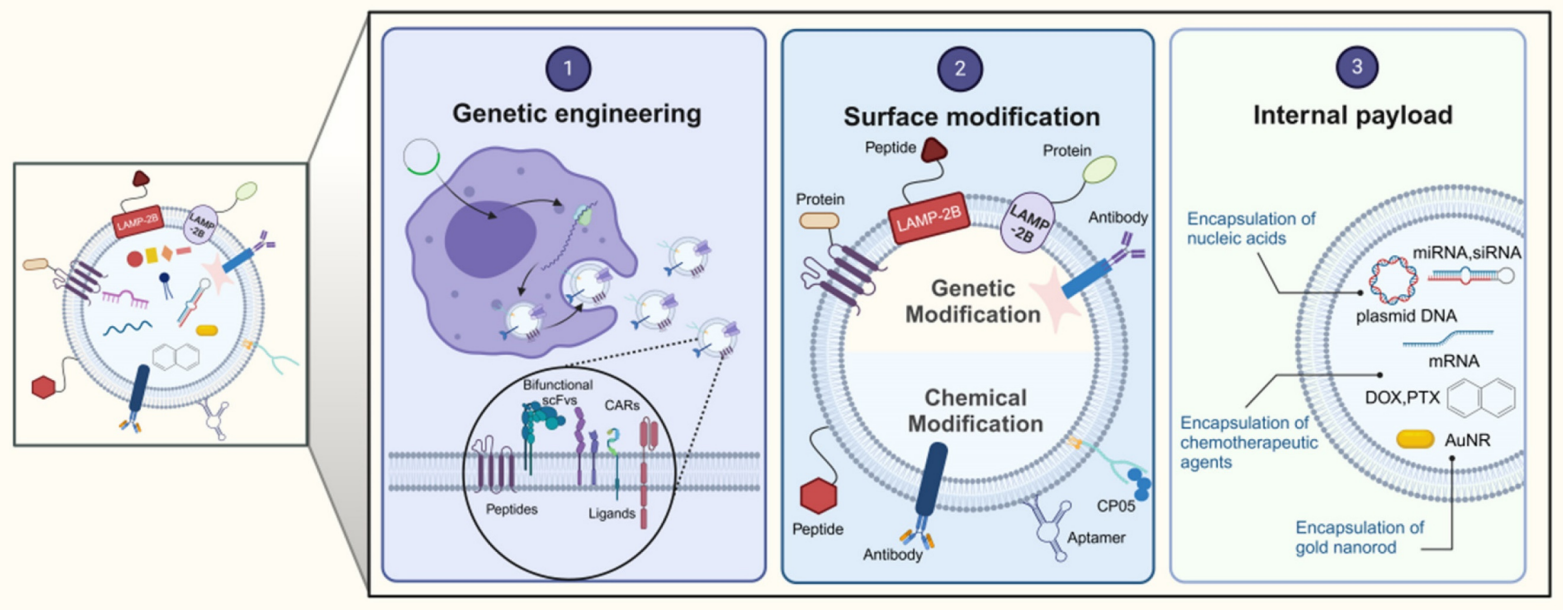
Strategies for the engineering EVs. The data were created using BioRender com.

EVs are secreted by a variety of cell types, and their functionality is contingent upon the cell type from which they originate. For example, EVs from cancer cells may carry miRNAs that foster tumorigenesis, whereas those from antigen-presenting cells (APCs) display major histocompatibility complex (MHC) class II proteins on their surface 24-27. Conversely, stem cell-derived EVs are enriched with therapeutic cargo, offering potential treatments for conditions such as osteoarthritis, acute lung injury, and neurodegenerative diseases 28,29. However, the inherent composition of natural EVs is inherently linked to their parent cell type 25, which presents certain limitations. To transcend these constraints, EVs can be engineered beyond their initial biogenic cargo to include desired components 30. This augmentation of EV cargo is termed "abundant supplementation," broadening the scope of EV applications. There are three principal strategies for EV modification: genetic engineering, surface modification, and internal loading (**Figure 2**). Genetic engineering involves altering the EV membrane to express specific proteins. Surface modification involves the targeted attachment of molecules to the EV surface for precise delivery. Internal loading refers to the encapsulation of exogenous substances within the EVs. These strategies aim to enhance the therapeutic potency of the cargo, improve in vivo tracking, and increase the targeting precision of EVs 31. The subsequent sections will delve into these EV engineering methods, their therapeutic applications, and a thorough examination of their benefits and limitations.

### 3.1 Genetic engineering

Recombinant DNA technology facilitates the biological modification of EVs. It has been demonstrated that host cells can be genetically programmed to produce EVs with specific cargo on their surface or within their lumen 32. Both nonviral and viral vectors are utilized to modify host cells prior to EV secretion. The vector system must ensure the stable integration of genetic material into host cells without disrupting their native functions or triggering unwanted immune responses in recipient cells. Viruses are advantageous for this purpose, as they naturally exploit and manipulate host mechanisms 33. Commonly employed viruses for host cell modification include adenoviruses, lentiviruses, retroviruses, adeno-associated viruses (AAVs), and herpes simplex viruses (HSVs), with retroviral and adenoviral systems being the most prevalent 34. This biological approach to genetic engineering of EVs is efficient and devoid of unnecessary side effects, maintaining the structural integrity of the EV membrane and broadening their utility across various applications, including fluorescence imaging, drug delivery, and targeting. However, there are limitations to this method. Firstly, it is restricted to proteins and peptides that can be expressed by the genetic material encapsulated within the engineered EVs 25. Secondly, if the protein of interest is cytotoxic, its overexpression may impair the viability of the host cells or induce apoptosis. Thirdly, the process of designing EVs through genetic engineering is labor-intensive and requires substantial infrastructure. Despite these challenges, cell bioengineering for the production of engineered EVs has garnered considerable interest, with the development of EVs characterized by high stability, drug solubility, and bioavailability representing the cutting-edge direction of this field 35-39.

### 3.2 Surface modification

Extracellular vesicles (EVs) can be engineered to enhance their targeting efficiency to cancer cells through two primary methods: transgenic expression and chemical attachment. Transgenic expression is achieved by integrating the coding sequence of a ligand with that of a signal peptide and a specific membrane protein, enabling EVs to present both targeted homing peptides and ligands on their surface. For example, EVs engineered to display the RVG peptide can effectively deliver opioid receptor siRNA to the brain 40. This approach exemplifies the potential of modifying EVs for precise therapeutic delivery.

The chemical approach to EV modification entails the direct attachment of molecules to the EV surface via covalent bonds. Copper-catalyzed azide-alkyne cycloaddition (CuAAC) stands out as an efficient technique for affixing both small molecules and large biologics to the EV surface 41. Noncovalent methods can also be employed to modify the EV surface. For instance, Kazunari Akiyoshi's group demonstrated that the use of electrostatic interactions, such as combining cationic lipids with the EV surface, can confer a positive surface charge to EVs, thereby enhancing their uptake 42. Despite the progress in targeting EVs through surface modification, challenges for clinical translation persist, including cytotoxicity, liver clearance, and tumor targeting efficiency, which must be addressed to ensure the efficacy of these methods in clinical practice.

### 3.3 Internal payload

The transportation of drugs or therapeutic molecules via extracellular vesicles (EVs) can be achieved through various loading methods, including incubation, electroporation, ultrasonication, extrusion, freeze-thaw cycles, and saponin-assisted loading 25,43-46. The choice of loading method is influenced by the drug's nature, with hydrophobic compounds being more readily incorporated into EVs through co-incubation, while hydrophilic molecules exhibit reduced diffusion efficiency across the lipid bilayer of EVs. Consequently, there is a need for novel physical loading techniques. A study by Haney et al. demonstrated the comparative loading efficiencies of catalase into EVs using different methods, with the ranking being: incubation < freeze-thaw cycles < ultrasonication < extrusion < electroporation 47. While electroporation and ultrasonication can enhance drug loading efficiency, they also carry the risk of damaging the EV membrane and causing RNA aggregation. Extrusion, in particular, can induce cellular toxicity, potentially due to alterations in the EV membrane structure. For instance, Fuhrmann et al. reported changes in the zeta potential of EVs after loading them with porphyrins through extrusion 48. Beyond the aforementioned methods, cellular nanoporation has emerged as a technique for generating a substantial quantity of EVs laden with therapeutic mRNAs and targeting peptides. Cellular nanoporation can increase the production of EVs, even from cells with low basal secretion rates, by up to 50 times, with a more than 1000-fold increase in EV mRNA transcripts. In conclusion, while traditional loading methods are established, there is a continued need for improvement in terms of loading efficiency and adaptability to different drug types. This quest for enhancement represents a frontier for future technological innovation in the field of EV-based therapeutics.

## 4. The potential of MSC-EVs in organ regeneration

Surgical resection for solid tumors, while common, faces inherent limitations. The chronic inflammatory state at the tumor site and the extensive damage to healthy tissue from surgery hinder the regeneration of healthy tissue. Thus, reconstructing healthy tissue post-tumoral excision presents a significant challenge. Extracellular vesicles (EVs) from mesenchymal stem cells (MSCs) significantly influence immune regulation by modulating signaling pathways across various tissues. These MSC-EVs have the capacity to mitigate or postpone tissue damage, enhance matrix remodeling, and promote tissue regeneration 29,49-52. The therapeutic impact of MSC-EV therapy is largely contingent upon the specific cargo they carry, which can stimulate or suppress various biomedical protein factors 53. In the following section, we will explore the therapeutic applications of MSC-EVs for tissue repair in a range of conditions affecting the heart, bones, cartilage, kidneys, liver, and skin 54. We will emphasize the common functional components of these EVs to provide a theoretical foundation for the reconstruction of healthy tissue following the resection of solid tumors.

### 4.1 Heart

**Figure 3:**
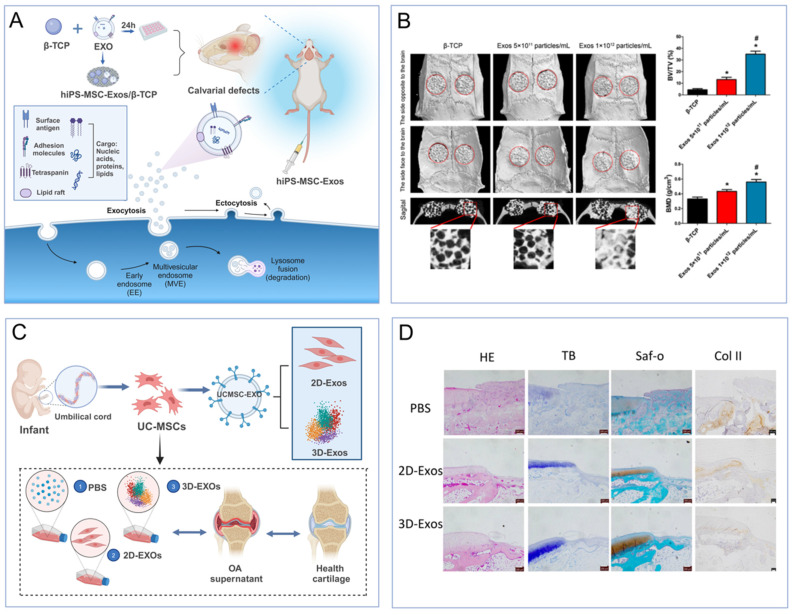
(A-B). MSC-EVs increase ATP levels, decrease oxidative stress and activate PI3K/Akt pathway to enhance myocardial viability and prevent adverse remodeling after myocardial ischemia/reperfusion injury 69. (C-D). Effects of 2D-EVs and 3D-EVs on matrix synthesis and the phenotypic stability of chondrocytes 79. The data were created using BioRender.com.

The process of cardiac regeneration post-injury is inherently slow, relying on the limited self-replication of existing myocardial cells and the recruitment and differentiation of resident cardiac stem cells 55. To augment this intrinsic healing capacity, MSC-EVs have been employed in cell-free therapeutic strategies. Specifically, human embryonic-derived MSC-EVs have demonstrated the ability to diminish infarct size in a mouse model of myocardial ischemia/reperfusion injury. This beneficial effect is mediated through the activation of the PI3K/Akt signaling pathway, which enhances myocardial viability and curbs adverse remodeling (**Figure 3A-B**) 56. To enhance this reparative function, strategies for EV engineering have been devised. Human umbilical cord-derived MSCs were transfected with the Akt gene, resulting in EVs that were highly enriched with this protein. These Akt-enriched EVs, when compared to their unmodified counterparts, significantly boosted in vitro endothelial cell proliferation, migration, and tube formation, as well as in vivo angiogenesis 57.

Studies have shown that the treatment methods of EVs can significantly influence their therapeutic impact. For example, hypoxic preconditioning of human bone mesenchymal stem cells (BMMSCs) enhances their in vitro bioactivity and improves the therapeutic efficacy of cynomolgus monkey BMMSCs for myocardial infarction (MI) in vivo 58,59. The hypoxic condition also augments the therapeutic potential of secreted EVs. Human BMMSC-EVs subjected to hypoxia induced more robust cardiac regeneration in a rat MI model compared to those isolated under normoxic conditions, attributed to enhanced angiogenesis at the infarct border zone 60. Moreover, hypoxia-modulated BMMSC-EVs from mice and rats, enriched with miR-125b-5p-EVs and miR-210-EVs, mitigated cardiomyocyte apoptosis by inhibiting pro-apoptotic genes p53 and BAK1, and by increasing the recruitment of cardiac progenitor cells to the infarcted area 61,62. Thus, EVs are pivotal in cardiac repair. Additionally, combining EVs with materials like hydrogels can further amplify their therapeutic effects. For example, encapsulating MSC-EVs within functional peptide hydrogels for delivery to cardiac defects allows for a sustained release and promotes superior cardiac regeneration. These EV/hydrogel combinations reduce inflammation, fibrosis, and apoptosis while fostering neovascularization around the infarct area in a rat MI model 53,63. Consequently, the regenerative capacity of EVs post-cardiac injury offers innovative solutions for addressing heart damage in the postoperative care of solid tumors.

### 4.2 Bone

The utilization of extracellular vesicles (EVs) from mesenchymal stem cells (MSCs) is an emerging strategy for bone regeneration. Researchers are exploring the potential of MSCs sourced from various origins to facilitate bone repair post-injury. Evidence suggests that EVs derived from human bone marrow MSCs (BMMSCs) and human induced pluripotent stem cells (hiPSCs) can foster bone formation and vascularization in rats with critical-sized femoral defects, and also promote the osteogenic differentiation of BMMSCs in vitro 64,65. Human BMMSC-EVs have been successfully modified with dimethyl malonate glycine to augment angiogenesis via the Akt/mTOR pathway. Additionally, the therapeutic potency of EVs has been enhanced by preconditioning human adipose-derived MSCs (ADMSCs) with the cytokine TNF-α, leading to increased proliferation of osteoblastic cells and osteogenic differentiation in vitro 66.

To enhance the therapeutic impact of EVs, they have been integrated into tissue engineering scaffolds. Human adipose-derived MSC-EVs (ADMSC-EVs) were immobilized on poly(lactic-co-glycolic acid) scaffolds enriched with biotin, thereby enhancing scaffold efficacy and promoting bone healing. In vitro studies demonstrated superior osteoconductivity for both bone marrow MSCs (BMMSCs) and osteoblasts when cultured on EV-functionalized scaffolds compared to unmodified ones. Corresponding in vivo studies utilizing a mouse bone defect model revealed significantly increased bone tissue and mature collagen formation 67,68. Additionally, human BMMSC-EVs loaded onto tricalcium phosphate scaffolds were found to promote healing in calcium-deficient bone by activating the PI3K/Akt signaling pathway, while rat BMMSC-EVs encapsulated within a decellularized bone matrix scaffold stimulated bone regeneration by enhancing graft vascularization (**Figure 3A-B**) 69. In summary, EVs exhibit robust tissue repair capabilities following bone injury, offering a novel therapeutic avenue for bone healing in the postoperative management of solid tumors.

### 4.3 Cartilage

Extracellular vesicles (EVs) from mesenchymal stem cells (MSCs) offer a promising cell-free therapeutic approach for cartilage injuries and osteoarthritis (OA) 36. Human bone marrow MSC-derived EVs have demonstrated the capacity to stimulate cartilage regeneration, evidenced by the increased production of type II collagen and proteoglycans in chondrocytes from OA patients. These components are integral to the extracellular matrix (ECM) and are essential for effective cartilage repair 70. Osteoarthritis is commonly linked to cartilage degradation, a process primarily driven by Wnt5A, which activates matrix metalloproteinases and diminishes ECM formation 71. Notably, human bone marrow MSC-EVs enriched with miR-92a-3p have been shown to counteract cartilage degradation and enhance repair both in vitro and in an OA mouse model by specifically targeting Wnt5A 72,73. In a separate study, preconditioning rat MSCs with transforming growth factor-beta (TGFβ) led to an increase in miR-135b within the EVs, which in turn stimulated chondrocyte proliferation and regulated cartilage repair via the specific protein 1 (Sp1) in an OA rat model 74. Furthermore, the application of human embryonic MSC-EVs in rat and mouse models of osteochondral defects has revealed coordinated mechanisms that facilitate osteochondral regeneration, including enhanced chondrocyte proliferation, reduced apoptosis, immune modulation, balanced ECM synthesis and degradation, and the restoration of matrix homeostasis 75-78.

Beyond loading EVs with specific miRNAs, alternative approaches have amplified the potency of MSC-EVs in cartilage restoration. Specifically, the three-dimensional culture of umbilical cord MSCs (UCMSCs) has yielded a higher output of EVs and more pronounced therapeutic outcomes in a rabbit model of cartilage defects, outperforming the conventional two-dimensional MSC-EVs cultivation method (**Figure 3C-D**) 79. Human iPSC-EVs, when integrated with in situ hydrogels, have been shown to effectively retain MSC-EVs at cartilage injury sites 80. This cell-free tissue patch, when combined with the native cartilage matrix, facilitates cellular deposition at the defect site, thereby enhancing functional cartilage repair.

3D printing technology has been harnessed to fabricate intricate structures with exceptional precision 81. Bone marrow MSC-derived EVs have been incorporated into cartilage ECM/gelatin methacrylate hydrogels, serving as bioinks for bioprinting applications. The 3D-printed constructs not only ensure the precise delivery of EVs but also avert mitochondrial dysfunction in degenerate cartilage cells in vitro and foster cartilage regeneration in a rabbit osteochondral defect model in vivo 82. Collectively, these findings underscore the robust tissue repair capabilities of EVs post-bone injury, heralding a novel therapeutic avenue for bone injury management following solid tumor surgery.

### 4.4 Kidney

**Figure 4:**
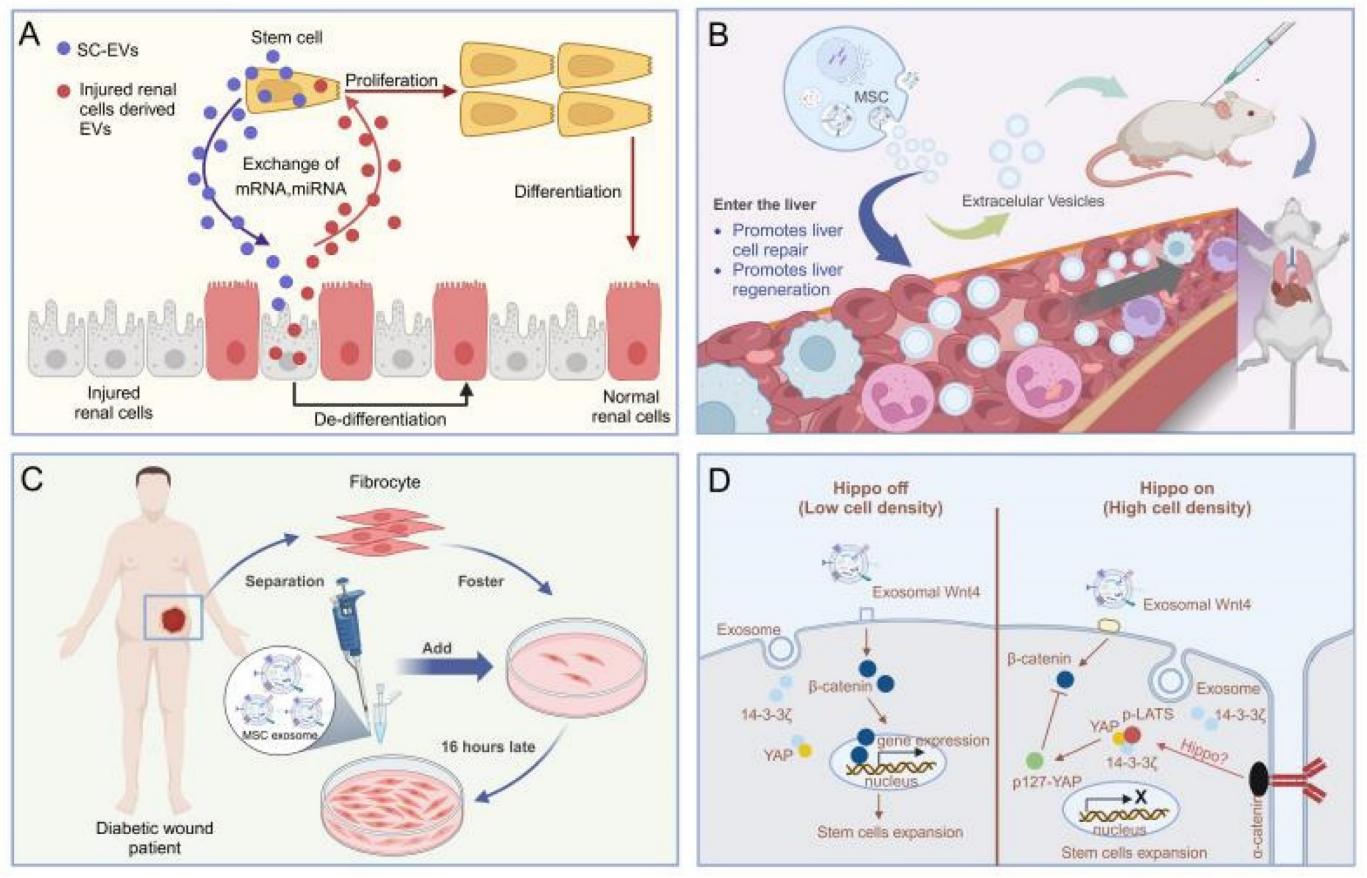
(A). MSC-EVs based therapy against acute kidney injury 84,85. (B). MSC-EVs promote hepatic regeneration in drug-induced liver injury models 88. (C). The MSC-EVs enhance the migration of normal and diabetic wound fibroblasts 98. (D). HucMSC EV-delivered 14-3-3ζ control of the wnt response during cutaneous regeneration 98,99. The data were created using BioRender.com.

The application of MSC-EVs for acute kidney injury (AKI) and chronic kidney disease (CKD) is emerging as a promising strategy for renal regeneration 83. Evidence suggests that MSC-EVs expedite the healing of injured tubular cells by enhancing cell proliferation, inhibiting apoptosis, and aiding in functional recuperation in glycerol-induced AKI. Mechanistic insights indicate that MSC-EVs transfer RNA molecules, including mRNAs and miRNAs, to impaired renal cells, thereby exerting anti-inflammatory, antiapoptotic, antifibrotic, and proangiogenic properties 84,85. (**Figure 4A**). A recent study has highlighted the influence of extracellular vesicles (EVs) on human tubular epithelial cells exposed to cisplatin. It has been observed that EVs upregulate the expression of antiapoptotic genes, such as B-cell lymphoma 2 and baculoviral IAP repeat containing 8, while downregulating genes associated with the execution phase of apoptosis, including caspase-1, caspase-8, and lymphotoxin-α 86. Additionally, MSC-EVs from diverse tissues have been scrutinized for their renal regenerative potential. EVs sourced from the human umbilical cord, Wharton's jelly, liver, and glomerular MSCs have demonstrated efficacy in facilitating recovery post-AKI 87. These EVs enhance tubular cell proliferation and mitigate inflammation and apoptosis via mitochondrial fission modulation. In a specific study, mouse bone marrow MSC-EVs encapsulated in self-assembling peptide nanofiber hydrogels were released at AKI sites in mouse models following ischaemia-reperfusion, leading to markedly enhanced therapeutic outcomes and improved renal function 87. The burgeoning research into MSC-EVs for kidney regeneration underscores the high promise of this approach, suggesting its potential as a strategy for postoperative renal repair in the context of renal cancer.

### 4.5 Liver

Several studies indicate that MSC-EVs may offer therapeutic benefits for liver disease treatment. For example, in a mouse model of liver injury induced by carbon tetrachloride (CCl4), human embryonic MSC-EVs have been shown to foster liver regeneration by enhancing hepatocyte proliferation and mitigating their apoptosis (**Figure 4B**) 88. In a rat model of liver ischemia-reperfusion injury, iPSC-EVs have been demonstrated to promote liver regeneration by inhibiting hepatocyte apoptosis, dampening the inflammatory response, and alleviating oxidative stress 89. Furthermore, research has indicated that human iPSC-EVs possess the capacity to stimulate liver cell proliferation both in vitro and in vivo by activating the sphingosine kinase and sphingosine-1-phosphate signaling pathways 90.

Similarly, UCMSC-EVs have been demonstrated to ameliorate liver function by mitigating oxidative stress and curtailing neutrophil infiltration, thus averting liver apoptosis 91. To augment the therapeutic efficacy of EVs, human embryonic MSC-EVs have been embedded within PEG hydrogels for a sustained release to the liver. In a rat model of chronic liver fibrosis, this strategy proved superior to conventional EV injections, exhibiting potent antiapoptotic, antifibrotic, and regenerative effects 92. Collectively, the burgeoning research on MSC-EVs for liver regeneration signals a highly promising approach. Consequently, the application of MSC-EVs for postoperative liver repair following cancer surgery is anticipated to be a promising strategy.

### 4.6 Skin

Wound healing is a multifaceted process encompassing a spectrum of cellular and molecular activities, such as cell migration, proliferation, angiogenesis, extracellular matrix (ECM) deposition, and tissue remodeling. Effective wound healing necessitates a sequential transition through phases of homeostasis, inflammation, proliferation, and remodeling. Improper healing can lead to excessive scarring 93. Studies have indicated that extracellular vesicles (EVs) from MSCs are instrumental in facilitating the healing of chronic wounds. A notable study disclosed that bone marrow MSC-derived EVs (BMMSC-EVs) markedly boosted fibroblast proliferation and migration in both healthy individuals and those with chronic wounds, as well as fostered endothelial cell angiogenesis (**Figure 4C**) 94. Another study demonstrated that human iPSC-EVs accelerated skin wound healing by enhancing collagen synthesis and vascularization 95. Analogously, human ADMSC-EVs efficiently stimulated the production of collagen and elastin in light-damaged human dermal fibroblasts in vitro and expedited wound healing in a mouse skin incision model in vivo 96. In a comparative study on rat skin wounds, ADMSC-EVs outperformed BMMSC-EVs in healing efficacy 97. Additionally, in vivo studies have illustrated that human UCMSC-EVs advance the healing of secondary burn wounds by activating the Wnt/β-catenin signaling pathway, leading to increased dermal fibroblast proliferation, angiogenesis, and diminished apoptosis of skin cells 98, 99 (**Figure 4D**). The therapeutic advantages of EVs are attributed to the function of specific miRNAs, including miR-21, miR-23a, miR-125b, and miR-145 100. In conclusion, MSC-EVs are pivotal in immune modulation and skin tissue regeneration. These exosomes mirror the role of stem cells, exerting robust effects by modulating immune pathways, enhancing the migration and proliferation of skin cells, and curtailing cell apoptosis. Consequently, the application of MSC-EVs for postoperative skin repair is a viable strategy.

## 5. The potential of MSC-EVs in solid tumor therapy

### 5.1 Regulatory effect of MSC-EVs itself on tumor signalling pathways

**Figure 5:**
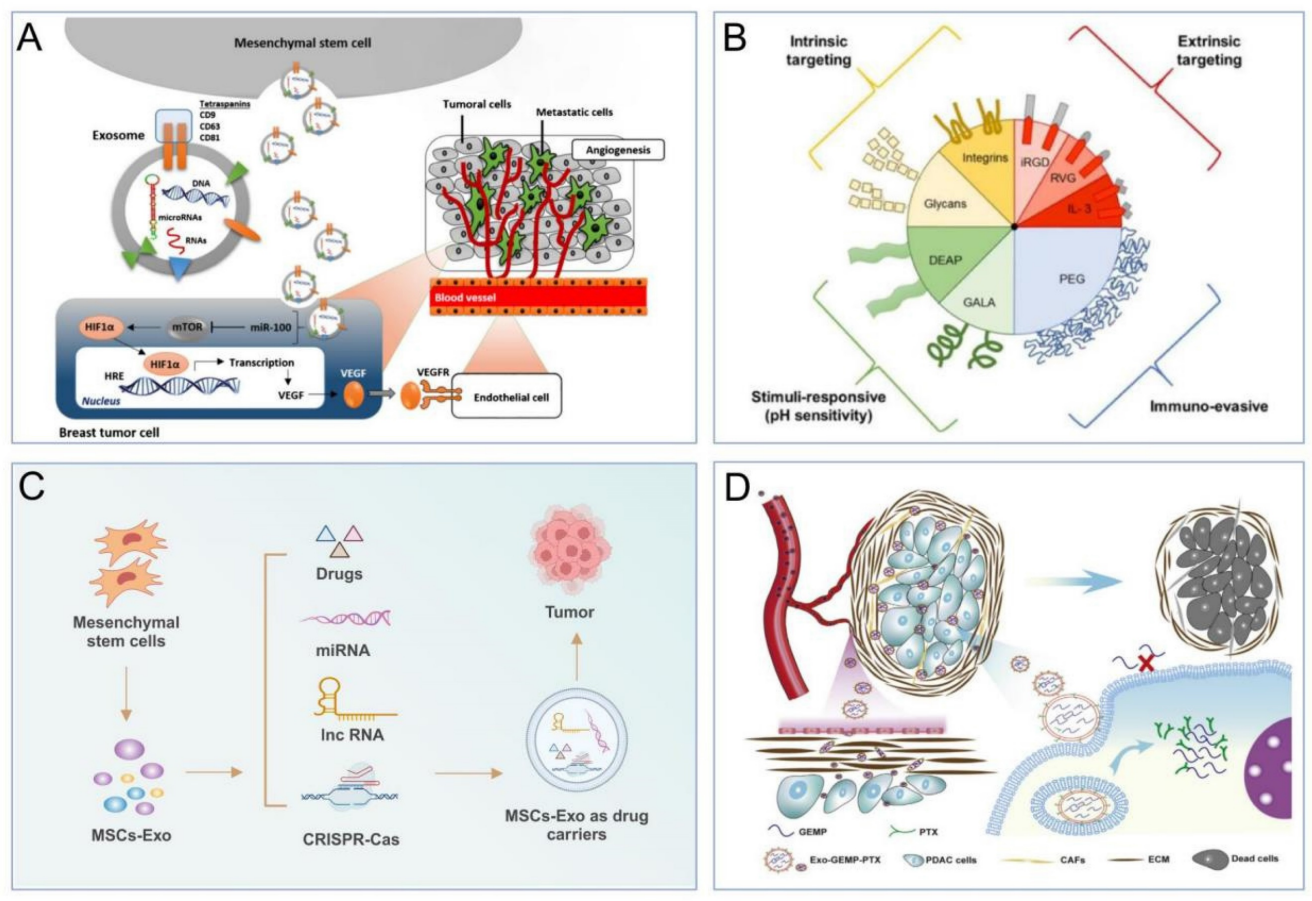
(A). EVs regulate tumor progression by affecting signalling pathway 102,103. (B). Examples of MSC-EVs with tumor-targeting ability that aid in drug delivery 115. (C). Drug delivery system of MSCs-EVs for tumor therapy. (D). BMSC-EVs loaded with PTX and GEMP for pancreatic cancer treatment 124.

The therapeutic potential of EVs extends beyond their critical role in sustaining normal physiological functions; they can also modulate tumor progression indirectly through various signaling pathways 101. For instance, MSC-EVs are enriched with microRNA-100, which can impede angiogenesis and curb the advancement of breast cancer by targeting the mTOR/HIF1A/VEGF signaling axis (**Figure 5A**) 102,103. Moreover, research indicates that miRNA-146b derived from MSC EVs can inhibit glial cell growth post-transplantation in rats, although the precise mechanisms remain to be elucidated 104. Likewise, BMMSC-EVs can impede the invasiveness, migration, and proliferation of pancreatic cancer cells by sequestering miR-338-5p and engaging the Wif1/Wnt8/β-catenin axis 105. Overall, the miRNAs delivered by MSC-EVs have the capacity to regulate tumor progression, heralding a novel paradigm for solid tumor therapies in the future.

### 5.2 Drug delivery of MSC-EVs

#### 5.2.1 Advantages of MSC-EVs as drug carriers

EVs derived from cells are superior to artificial nanocarriers for drug delivery due to their excellent biocompatibility, low immunogenicity, and high specificity for targets 106,107. Firstly, EVs are natural vesicles secreted by cells, and their structural and compositional similarities with cell membranes allow them to be easily tolerated by the body and evade phagocytosis by immune system cells 49,108. For instance, Tian et al. demonstrated that EVs loaded with doxorubicin showed low toxicity and were effective at targeting tumor tissues 109. Secondly, EVs have advantages such as deep tissue penetration and a prolonged circulation half-life 110. Thirdly, the presence of phospholipids, signalling transduction factors, and adhesion factors on EV membranes contributes to their fusogenic capability with target cells, enabling them to traverse biological barriers, such as the blood‒brain barrier 111. The use of MSC-EVs as a drug carrier is attributed to the fact that MSC-EVs has unique advantages over other cell types. For example, first of all, MSCs have a greater capacity to secrete EVs. In the meantime, their low immunogenicity enables them to evade immune activation and clearance by the human immune system. Secondly, MSCs have the ability to target tumors, so MSC-derived EVs exhibit strong tumor-targeting ability (**Figure 5B**) 112-115. Furthermore, their small size enhances tumor permeability and retention, allowing them to selectively accumulate at the disease site 116,117. The solid lipid bilayer structure of MSC-derived EVs protects their cargo from the harsh TME and prevents the cell engulfment-lysosomal pathway 118. These advantages make MSC-derived EVs an ideal drug carrier for the treatment of solid tumors 115. In the next section, we will discuss the specific applications of MSC-derived EVs as a drug delivery system.

#### 5.2.2 Applications of MSC-EVs as drug carriers

MSC-EVs exhibit significant potential in the delivery of therapeutic drugs for cancer treatment (**Figure 5C**) 119. They enhance the inhibitory impact on tumor growth and the precision of drug delivery to tumor sites, surpassing traditional chemotherapy methods. Studies have demonstrated that MSC-derived EVs loaded with DOX can effectively suppress the proliferation of osteosarcoma cells in vitro with higher efficacy and lower cytotoxicity compared to free DOX 120. Likewise, these DOX-loaded MSC-EVs have efficiently targeted MUC1-positive colorectal cancer cells in vitro 121. In vivo studies have shown that DOX delivered by MSC-EVs accumulates highly at the tumor site, significantly inhibiting tumor growth while reducing systemic toxicity and liver clearance. Research indicates that mouse BMSCs can be loaded with PTX through in vitro exposure to high doses of PTX 122. These PTX-loaded BMSCs can then secrete EVs rich in PTX, exerting potent antiproliferative effects on pancreatic cancer cells. MSC-EVs loaded with PTX have been shown to inhibit tumor growth and metastasis more effectively than free PTX, at a 1000-fold reduced dose 123. Recent studies have highlighted that BMSC-derived EVs loaded with PTX and gemcitabine (GEMP) display exceptional homing and penetration capabilities for pancreatic cancer treatment, both in vitro and in vivo, with significantly enhanced antitumor efficacy (**Figure 5D**) 124. Honokiol, a versatile compound with novel antineoplastic properties, has been effectively encapsulated into MSC-EVs using ultrasound methods 125, demonstrating superior antitumor effects through efficient cellular uptake compared to free honokiol. Norcantharidin, a demethylated derivative of cantharidin with potent anticancer activity and minimal side effects, has been shown to exert significant anticancer effects when delivered via BMSC-derived EVs, promoting homing to the tumor site without systemic toxicity in hepatocellular carcinoma treatment 126. Moreover, norcantharidin-loaded BMSC-EVs have been observed to repair damaged liver tissue by promoting cell proliferation and reducing hepatocellular oxidative stress 127. In summary, MSC-derived EVs, with their robust tumor-homing capabilities, emerge as promising platforms for targeted antitumor drug delivery. However, the clinical application of MSC-EVs in cancer therapy requires further research to expedite its therapeutic potential.

#### 5.2.3 Sensitizing effects of MSC-EVs on tumor drugs

**Figure 6:**
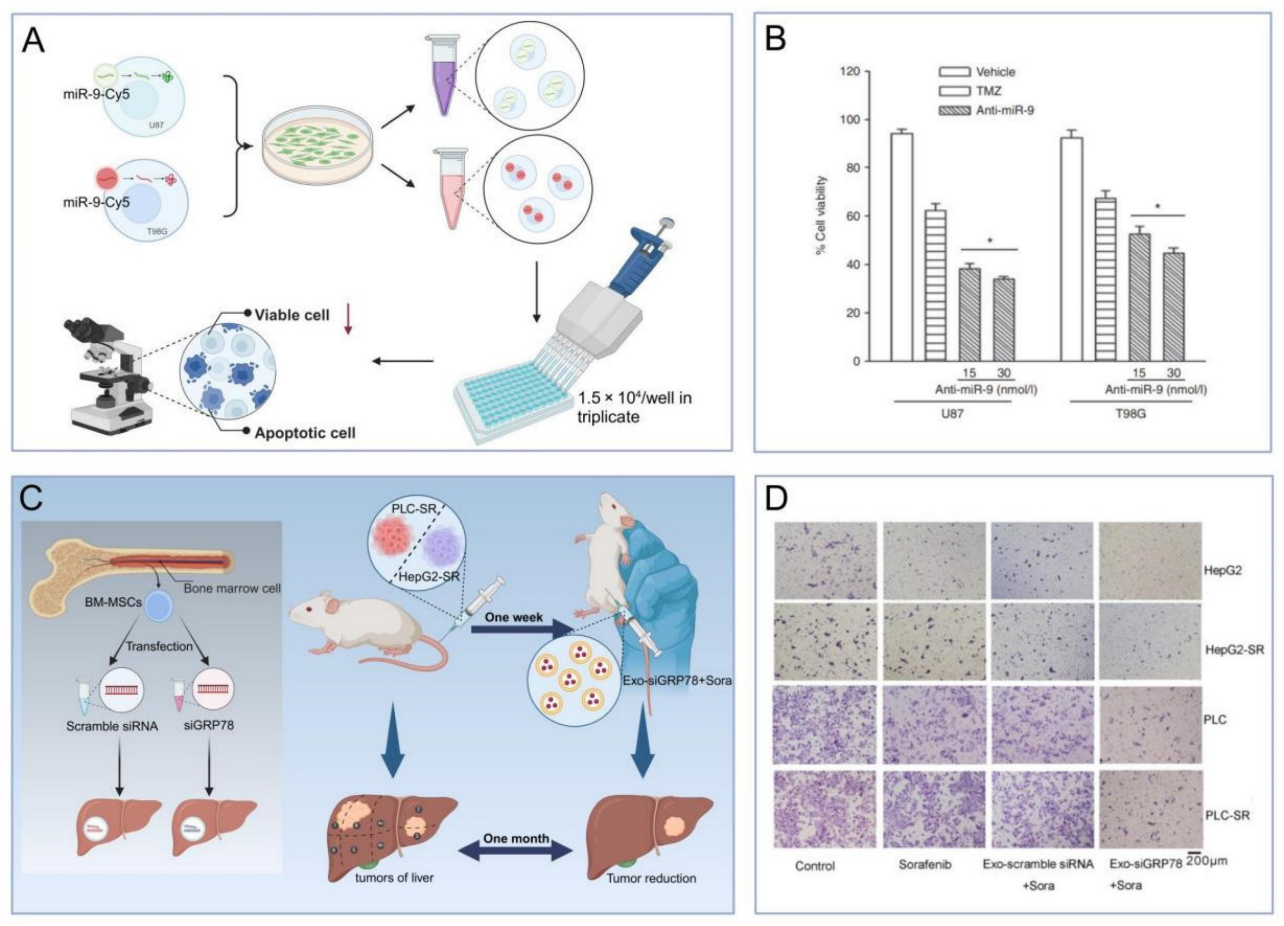
(A-B). Anti-miR-9 treatment enhanced TMZ-induced cell death 130. (C-D). The effect of siGRP78-modified EVs on the metastasis of sorafenib-resistant cancer cells 131. The data were created using BioRender.com.

Recent studies suggest that MSC-EVs, beyond their role as drug carriers, have the potential to augment cancer therapy. Specifically, EVs from miR-199-modified ADMSCs have increased the sensitivity to DOX in hepatocellular carcinoma by inhibiting the mTOR signaling pathway both in vitro and in vivo 128. In glioblastoma, miR-199a has been shown to curb the proliferation, invasion, and migration of cells in both in vitro and in vivo settings 129. MSC-EVs overexpressing miR-199a have demonstrated the ability to impede glioblastoma progression by suppressing AGAP2 expression and increasing sensitivity to temozolomide (TMZ). Moreover, EVs from MSCs transfected with anti-miR-9-Cy5 have mitigated TMZ resistance in glioblastoma cells by enhancing caspase activity and inducing cell death in response to TMZ. The delivery of small interfering RNA (siRNA) via MSC-derived EVs represents another promising therapeutic approach for boosting drug sensitivity across various cancers (**Figure 6A-B**) 130. In hepatocellular carcinoma, BMSC-derived EVs modified with GRP7-siRNA have sensitized cells to sorafenib, which, when combined with si-GRP78-modified BMSC-derived EVs, has shown to inhibit the growth and invasion of hepatocellular carcinoma cells in vitro (**Figure 6C-D**) 131. Thus, the drug-sensitizing effect of MSC-EVs is set to amplify their tumoricidal impact, offering substantial promise for postoperative management of solid tumors.

## 6. Conclusion

**Figure 7:**
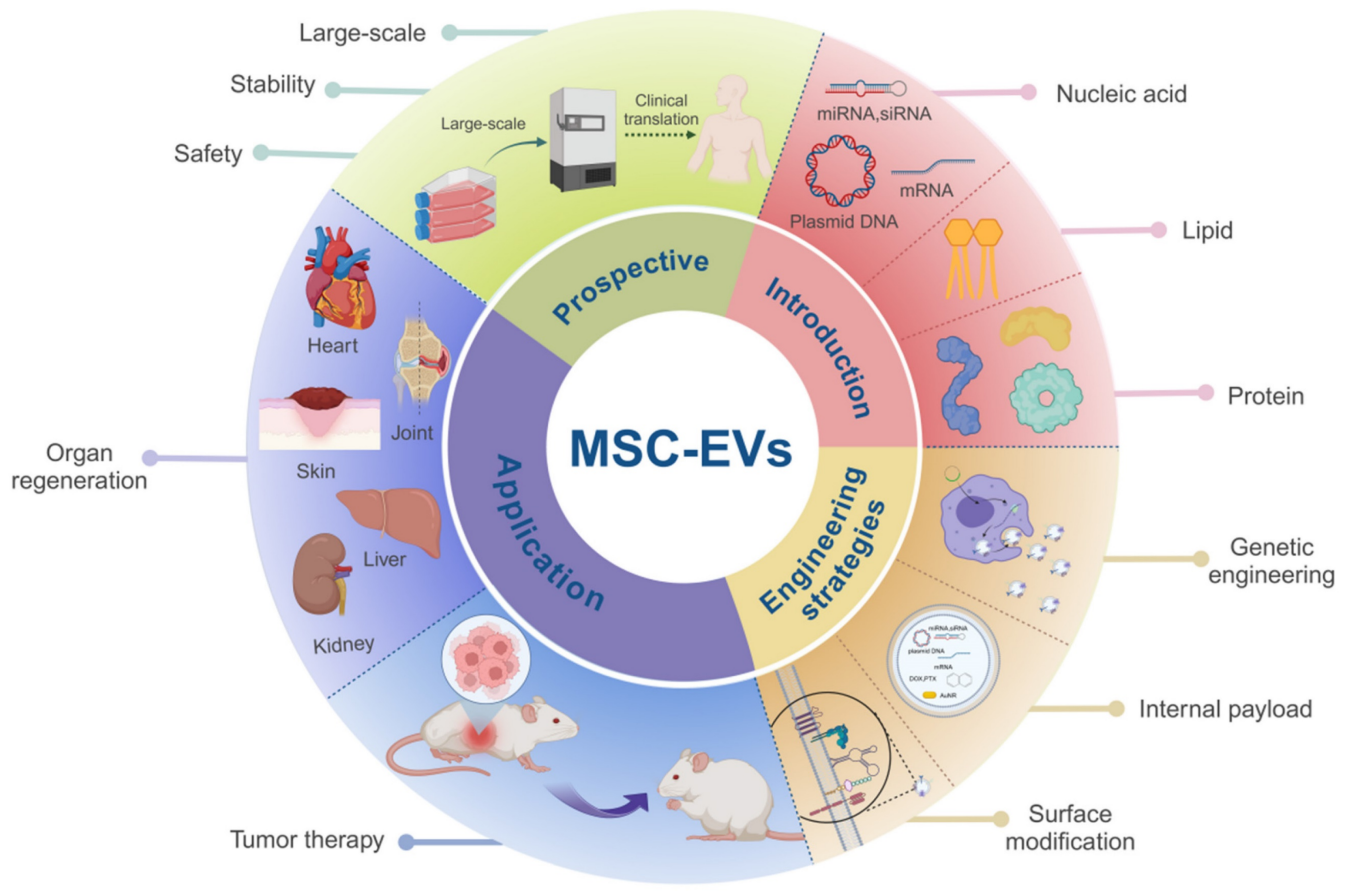
Summary of MSC-EVs in soild tumor treatment. MSC-EVs composed of different materials have been developed using various engineering strategies. After optimization, MSC-EVs can deliver active substances, and their own active substances can prevent tumor recurrence and promote tissue repair. Future studies should focus on standard quality specifications during production to ensure purity, safety and economy for mass production and clinical applications. The data were created using BioRender.com.

In this review, we aim to provide an overview of solid tumor treatment via MSC-EVs, which include organ regeneration and tumor suppression. We compared the benefits and drawbacks of MSC-EVs and examined the correlation between their biological features and engineering to provide guidance on their use in solid tumor therapy. Furthermore, we discussed the challenges associated with the utilization of MSC-EVs, future applications, and potential strategies and design principles that could enhance their effectiveness in tumor suppression and tissue regeneration (**Figure 7**).

There is an inherent relationship and tension between oncological treatment and tissue repair. The objective of tumor therapy is to eradicate or manage cancer cells, curbing tumor growth and metastasis, thereby addressing the malignancy. Conversely, tissue repair denotes the restitution and reconstruction of the function and architecture of compromised tissues or organs through a spectrum of physiological and biological mechanisms post-injury. Common modalities in oncology, such as surgery, radiotherapy, chemotherapy, and targeted therapies, may inadvertently harm healthy tissues and cells. Specifically, radiotherapy and chemotherapy can exert toxic side effects on the body's normal tissues and organs, precipitating tissue damage and impaired function. This dynamic underscores the dichotomy between cancer treatment and tissue restoration: in combating tumors, a certain level of injury to healthy tissues is often inevitable, potentially compromising their capacity for healing and regeneration. Moreover, tissue repair and regeneration encompass intricate biological processes hinged on the orchestrated activities of cell proliferation, differentiation, and migration. Disruptions to these processes during cancer treatment could impede the natural restoration and reconstitution of damaged tissues, risking the loss or deterioration of tissue functionality. Hence, a balanced approach is imperative in oncological practice, weighing the therapeutic benefits against the collateral damage to healthy tissues to safeguard the innate reparative and regenerative capabilities of the tissue. In essence, the interplay and conflict between tumor treatment and tissue repair must be judiciously navigated. It is essential to holistically assess the therapeutic efficacy and its repercussions on tissue repair, selecting apt treatment strategies to optimize the preservation of tissue repair and regeneration, thereby ensuring optimal patient outcomes.

Numerous studies have posited the beneficial role of MSC-EVs in connective tissue regeneration and oncology. Nonetheless, caution is warranted regarding the potential risks associated with their therapeutic use. Unaltered MSC-EVs could potentially foster tumor progression, underscoring the need for careful design and optimization to ensure safety and effectiveness. In conclusion, while MSC-EVs show considerable potential in both applications, ongoing research is essential to overcome the challenges posed by their integrated use. The clinical deployment of MSC-EV treatments faces notable challenges: 1. Technical Limitations: Achieving high-purity and high-yield MSC-EVs is challenging, necessitating advancements in purification and production technologies to fulfill clinical demands. 2. Substance-Effect Correlation: The therapeutic impact of specific components within MSC-EVs, such as lipids, proteins, and nucleic acids, remains unclear. Further research is required to understand the efficacy, safety, and optimal dosage of these endogenous substances when utilized therapeutically. 3. Targeting Specificity: The nonspecific targeting of MSC-EVs could lead to unintended side effects. Given their ability to engage multiple cell types, the mechanism of receptor selection in complex in vivo settings is not well understood, underscoring the need to enhance the specificity of MSC-EV targeting. 4. Drug Delivery Efficiency: As drug carriers, the efficiency of drug loading into MSC-EVs while preserving their structural and functional integrity presents another challenge. Future studies must focus on developing methods to effectively encapsulate therapeutic agents. MSC-EV research is a burgeoning field, with ongoing technological and scientific advancements expected to shed light on the heterogeneity, functionality, and clinical potential of these EVs. Further development of related technologies and studies is essential to unlock the full therapeutic potential of MSC-EVs.

## Abbreviations

AAVs: adeno-associated viruses
ADMSCs: adipose-derived MSCs
AKI: acute kidney injury
APCs: antigen-presenting cells
ApoBDs: apoptotic bodies
BAK1: BCL2 antagonist/killer 1
BMMSCs: bone mesenchymal stem cells
CKD: chronic kidney disease
ECM: extracellular matrix
EVs: Extracellular vesicles
hiPSC: human induced pluripotent stem cell
HSPs: heat shock proteins
HSVs: herpes simplex viruses
ICAM-1: intercellular adhesion molecule 1
ILVs: intraluminal vesicles
MHC: major histocompatibility complex
MI: myocardial infarction
MSCs: Mesenchymal stem cells
MSC-EVs: Mesenchymal stem cell extracellular vesicles
MVBs: multivesicular bodies
RBPs: RNA-binding proteins
Sp1: specific protein 1
TGFβ: transforming growth factor-beta
TMZ: temozolomide
WHO: the World Health Organization

## References

[1] Bray F, Laversanne M, Weiderpass E, Soerjomataram I (2021). The ever-increasing importance of cancer as a leading cause of premature death worldwide. Cancer.

[2] Cao W, Chen H, Yu Y, Li N, Chen W (2021). Changing profiles of cancer burden worldwide and in China: a secondary analysis of the global cancer statistics 2020. Chin Med J (Engl).

[3] Sung H, Ferlay J, Siegel RL, Laversanne M, Soerjomataram I, Jemal A (2021). Global Cancer Statistics 2020: GLOBOCAN Estimates of Incidence and Mortality Worldwide for 36 Cancers in 185 Countries. CA: A Cancer Journal for Clinicians.

[4] Hou AJ, Chen LC, Chen YY (2021). Navigating CAR-T cells through the solid-tumour microenvironment. Nat Rev Drug Discov.

[5] Wagner J, Wickman E, DeRenzo C, Gottschalk S (2020). CAR T Cell Therapy for Solid Tumors: Bright Future or Dark Reality?. Mol Ther.

[6] He D, Li H (2020). Bifunctional Cx43 Mimic Peptide Grafted Hyaluronic Acid Hydrogels Inhibited Tumor Recurrence and Stimulated Wound Healing for Postsurgical Tumor Treatment. Adv Funct Mater.

[7] Riml S, Larcher L, Kompatscher P (2013). Complete excision of nonmelanotic skin cancer: a matter of surgical experience. Ann Plast Surg.

[8] Etzkorn JR, Sharkey JM, Grunyk JW, Shin TM, Sobanko JF, Miller CJ (2017). Frequency of and risk factors for tumor upstaging after wide local excision of primary cutaneous melanoma. J Am Acad Dermatol.

[9] van der Eerden PA, Lohuis PJFM, Hart AAM, Mulder WC, Vuyk H (2008). Secondary Intention Healing after Excision of Nonmelanoma Skin Cancer of the Head and Neck: Statistical Evaluation of Prognostic Values of Wound Characteristics and Final Cosmetic Results. Plast Reconstr Surg.

[10] Mateescu B, Kowal EJ, van Balkom BW, Bartel S, Bhattacharyya SN, Buzás EI (2017). Obstacles and opportunities in the functional analysis of extracellular vesicle RNA - an ISEV position paper. J Extracell Vesicles.

[11] Lener T, Gimona M, Aigner L, Börger V, Buzas E, Camussi G (2015). Applying extracellular vesicles based therapeutics in clinical trials - an ISEV position paper. J Extracell Vesicles.

[12] Cheng L, Hill AF (2022). Therapeutically harnessing extracellular vesicles. Nat Rev Drug Discov.

[13] Lai P, Weng J, Guo L, Chen X, Du X (2019). Novel insights into MSC-EVs therapy for immune diseases. Biomark Res.

[14] Li J, Jiang X, Wang K (2019). Exosomal miRNA: an alternative mediator of cell-to-cell communication. ExRNA.

[15] Dellar ER, Hill C, Melling GE, Carter DRF, Baena-Lopez LA (2022). Unpacking extracellular vesicles: RNA cargo loading and function. Journal of Extracellular Biology.

[16] Fabbiano F, Corsi J, Gurrieri E, Trevisan C, Notarangelo M, D'Agostino VG (2020). RNA packaging into extracellular vesicles: An orchestra of RNA-binding proteins?. J Extracell Vesicles.

[17] Zhang Z, Wang Z, Zhang C, Yao Z, Zhang S, Wang R (2024). Advanced Terahertz Refractive Sensing And Fingerprint Recognition Through Metasurface-Excited Surface Waves. Adv Mater.

[18] Hou YC, Zhang C, Zhang ZJ, Xia L, Rao KQ, Gu LH (2022). Aggregation-Induced Emission (AIE) and Magnetic Resonance Imaging Characteristics for Targeted and Image-Guided siRNA Therapy of Hepatocellular Carcinoma. Adv Healthc Mater.

[19] Zhang C, Xia D, Liu J, Huo D, Jiang X, Hu Y (2020). Bypassing the Immunosuppression of Myeloid-Derived Suppressor Cells by Reversing Tumor Hypoxia Using Platelet-Inspired Platform. Adv Funct Mater.

[20] Jenjaroenpun P, Kremenska Y, Nair VM, Kremenskoy M, Joseph B, Kurochkin IV (2013). Characterization of RNA in exosomes secreted by human breast cancer cell lines using next-generation sequencing. PeerJ.

[21] Qiu G, Zheng G, Ge M, Wang J, Huang R, Shu Q (2019). Functional proteins of mesenchymal stem cell-derived extracellular vesicles. Stem Cell Res Ther.

[22] Lai RC, Lim SK (2019). Membrane lipids define small extracellular vesicle subtypes secreted by mesenchymal stromal cells. J Lipid Res.

[23] Sagini K, Costanzi E, Emiliani C, Buratta S, Urbanelli L (2018). Extracellular Vesicles as Conveyors of Membrane-Derived Bioactive Lipids in Immune System. International Journal of Molecular Sciences.

[24] Joo HS, Suh JH, Lee HJ, Bang ES, Lee JM (2020). Current Knowledge and Future Perspectives on Mesenchymal Stem Cell-Derived Exosomes as a New Therapeutic Agent. Int J Mol Sci.

[25] Mishra A, Singh P, Qayoom I, Prasad A, Kumar A (2021). Current strategies in tailoring methods for engineered exosomes and future avenues in biomedical applications. J Mater Chem B.

[26] Lindenbergh M, Stoorvogel W (2018). Antigen Presentation by Extracellular Vesicles from Professional Antigen-Presenting Cells. Annu Rev Immunol.

[27] Xu M, Feng T, Liu B, Qiu F, Xu Y, Zhao Y (2021). Engineered exosomes: desirable target-tracking characteristics for cerebrovascular and neurodegenerative disease therapies. Theranostics.

[28] Shojaei S, Hashemi SM, Ghanbarian H, Salehi M, Mohammadi-Yeganeh S (2019). Effect of mesenchymal stem cells-derived exosomes on tumor microenvironment: Tumor progression versus tumor suppression. J Cell Physiol.

[29] Xunian Z, Kalluri R (2020). Biology and therapeutic potential of mesenchymal stem cell-derived exosomes. Cancer Sci.

[30] Liang Y, Duan L, Lu J, Xia J (2021). Engineering exosomes for targeted drug delivery. Theranostics.

[31] Li Y, Wei S, Li S, Zheng P (2024). Strategies and Challenges of Mesenchymal Stem Cells-Derived Extracellular Vesicles in Infertility. Tissue Eng, Part B.

[32] Zhang Y, Li Q, Liu X, Fan C, Liu H, Wang L (2020). Prescribing DNA Origami Patterns via Scaffold Decoration. Small.

[33] Koppers-Lalic D, Hogenboom MM, Middeldorp JM, Pegtel DM (2013). Virus-modified exosomes for targeted RNA delivery; a new approach in nanomedicine. Adv Drug Deliv Rev.

[34] Li Y, Tew SR, Russell AM, Gonzalez KR, Hardingham TE, Hawkins RE (2004). Transduction of passaged human articular chondrocytes with adenoviral, retroviral, and lentiviral vectors and the effects of enhanced expression of SOX9. Tissue Eng.

[35] Li Y, Lu H, Qu Z, Li M, Zheng H, Gu P (2022). Phase transferring luminescent gold nanoclusters via single-stranded DNA. Science China Chemistry.

[36] Pang L, Jin H, Lu Z, Xie F, Shen H, Li X (2023). Treatment with Mesenchymal Stem Cell-Derived Nanovesicle-Containing Gelatin Methacryloyl Hydrogels Alleviates Osteoarthritis by Modulating Chondrogenesis and Macrophage Polarization. Adv Healthc Mater.

[37] Li Y, Zhai T, Chen J, Shi J, Wang L, Shen J (2022). Water-Dispersible Gold Nanoclusters: Synthesis Strategies, Optical Properties, and Biological Applications. Chem. Eur. J.

[38] Zhang C, Jing X, Guo L, Cui C, Hou X, Zuo T (2021). Remote Photothermal Control of DNA Origami Assembly in Cellular Environments. Nano Lett.

[39] Cheng G, Liu X, Liu Y, Liu Y, Ma R, Luo J (2022). Ultrasmall Coordination Polymers for Alleviating ROS-Mediated Inflammatory and Realizing Neuroprotection against Parkinson's Disease. Research (Washington).

[40] Alvarez-Erviti L, Seow Y, Yin H, Betts C, Lakhal S, Wood MJ (2011). Delivery of siRNA to the mouse brain by systemic injection of targeted exosomes. Nat Biotechnol.

[41] Smyth T, Petrova K, Payton NM, Persaud I, Redzic JS, Graner MW (2014). Surface functionalization of exosomes using click chemistry. Bioconjug Chem.

[42] Mizuta R, Sasaki Y, Kawasaki R, Katagiri K, Sawada SI, Mukai SA (2019). Magnetically Navigated Intracellular Delivery of Extracellular Vesicles Using Amphiphilic Nanogels. Bioconjug Chem.

[43] Zhang C, Huang H, Chen J, Zuo T, Ou Q, Ruan G (2023). DNA Supramolecular Hydrogel-Enabled Sustained Delivery of Metformin for Relieving Osteoarthritis. ACS Appl Mater Interfaces.

[44] Zhang C, Yuan Y, Wu K, Wang Y, Zhu S, Shi J (2022). Driving DNA Origami Assembly with a Terahertz Wave. Nano Lett.

[45] Zhang C, Ren J, He J, Ding Y, Huo D, Hu Y (2018). Long-term monitoring of tumor-related autophagy in vivo by Fe(3)O(4)NO· nanoparticles. Biomaterials.

[46] Zhang C, Ren J, Hua J, Xia L, He J, Huo D (2018). Multifunctional Bi2WO6 Nanoparticles for CT-Guided Photothermal and Oxygen-free Photodynamic Therapy. ACS Appl Mater Interfaces.

[47] Fuhrmann G, Serio A, Mazo M, Nair R, Stevens MM (2015). Active loading into extracellular vesicles significantly improves the cellular uptake and photodynamic effect of porphyrins. J Control Release.

[48] Podolak I, Galanty A, Sobolewska D (2010). Saponins as cytotoxic agents: a review. Phytochem Rev.

[49] Huang H, Zhang C, Wang X, Shao J, Chen C, Li H (2020). Overcoming Hypoxia-Restrained Radiotherapy Using an Erythrocyte-Inspired and Glucose-Activatable Platform. Nano Lett.

[50] Sun Y, Hefu Z, Li B, Lifang W, Zhijie S, Zhou L (2023). Plasma Extracellular Vesicle MicroRNA Analysis of Alzheimer's Disease Reveals Dysfunction of a Neural Correlation Network. Research (Wash D C).

[51] Zuo H, Tao J, Shi H, He J, Zhou Z, Zhang C (2018). Platelet-mimicking nanoparticles co-loaded with W(18)O(49) and metformin alleviate tumor hypoxia for enhanced photodynamic therapy and photothermal therapy. Acta Biomater.

[52] Zhao P, Ren S, Liu Y, Huang W, Zhang C, He J (2018). PL-W18O49-TPZ Nanoparticles for Simultaneous Hypoxia-Activated Chemotherapy and Photothermal Therapy. ACS Appl Mater Interfaces.

[53] Tsiapalis D, O Driscoll L (2020). Mesenchymal Stem Cell Derived Extracellular Vesicles for Tissue Engineering and Regenerative Medicine Applications. Cells.

[54] Yan Z, Zhang T, Wang Y, Xiao S, Gao J (2023). Extracellular vesicle biopotentiated hydrogels for diabetic wound healing: The art of living nanomaterials combined with soft scaffolds. Mater Today Bio.

[55] Senyo SE, Steinhauser ML, Pizzimenti CL, Yang VK, Cai L, Wang M (2013). Mammalian heart renewal by pre-existing cardiomyocytes. Nature.

[56] Arslan F, Lai RC, Smeets MB, Akeroyd L, Choo A, Aguor EN (2013). Mesenchymal stem cell-derived exosomes increase ATP levels, decrease oxidative stress and activate PI3K/Akt pathway to enhance myocardial viability and prevent adverse remodeling after myocardial ischemia/reperfusion injury. Stem Cell Res.

[57] Ma J, Zhao Y, Sun L, Sun X, Zhao X, Sun X (2017). Exosomes Derived from Akt-Modified Human Umbilical Cord Mesenchymal Stem Cells Improve Cardiac Regeneration and Promote Angiogenesis via Activating Platelet-Derived Growth Factor D. Stem Cells Transl Med.

[58] Hu X, Wu R, Shehadeh LA, Zhou Q, Jiang C, Huang X (2014). Severe hypoxia exerts parallel and cell-specific regulation of gene expression and alternative splicing in human mesenchymal stem cells. BMC Genomics.

[59] Hu X, Xu Y, Zhong Z, Wu Y, Zhao J, Wang Y (2016). A Large-Scale Investigation of Hypoxia-Preconditioned Allogeneic Mesenchymal Stem Cells for Myocardial Repair in Nonhuman Primates. Circ Res.

[60] Bian S, Zhang L, Duan L, Wang X, Min Y, Yu H (2014). Extracellular vesicles derived from human bone marrow mesenchymal stem cells promote angiogenesis in a rat myocardial infarction model. Journal of Molecular Medicine.

[61] Zhu J, Lu K, Zhang N, Zhao Y, Ma Q, Shen J (2018). Myocardial reparative functions of exosomes from mesenchymal stem cells are enhanced by hypoxia treatment of the cells via transferring microRNA-210 in an nSMase2-dependent way. Artif Cells Nanomed Biotechnol.

[62] Zhu L, Tian T, Wang J, He J, Chen T, Pan M (2018). Hypoxia-elicited mesenchymal stem cell-derived exosomes facilitates cardiac repair through miR-125b-mediated prevention of cell death in myocardial infarction. Theranostics.

[63] Han C, Zhou J, Liang C, Liu B, Pan X, Zhang Y (2019). Human umbilical cord mesenchymal stem cell derived exosomes encapsulated in functional peptide hydrogels promote cardiac repair. Biomater Sci.

[64] Liang B, Liang J, Ding J, Xu J, Xu J, Chai Y (2019). Dimethyloxaloylglycine-stimulated human bone marrow mesenchymal stem cell-derived exosomes enhance bone regeneration through angiogenesis by targeting the AKT/mTOR pathway. Stem Cell Res Ther.

[65] Qi X, Zhang J, Yuan H, Xu Z, Li Q, Niu X (2016). Exosomes Secreted by Human-Induced Pluripotent Stem Cell-Derived Mesenchymal Stem Cells Repair Critical-Sized Bone Defects through Enhanced Angiogenesis and Osteogenesis in Osteoporotic Rats. Int J Biol Sci.

[66] Lu Z, Chen Y, Dunstan C, Roohani-Esfahani S, Zreiqat H (2017). Priming Adipose Stem Cells with Tumor Necrosis Factor-Alpha Preconditioning Potentiates Their Exosome Efficacy for Bone Regeneration. Tissue Eng Part A.

[67] Chen L, Mou S, Li F, Zeng Y, Sun Y, Horch RE (2019). Self-Assembled Human Adipose-Derived Stem Cell-Derived Extracellular Vesicle-Functionalized Biotin-Doped Polypyrrole Titanium with Long-Term Stability and Potential Osteoinductive Ability. ACS Appl Mater Interfaces.

[68] Li W, Liu Y, Zhang P, Tang Y, Zhou M, Jiang W (2018). Tissue-Engineered Bone Immobilized with Human Adipose Stem Cells-Derived Exosomes Promotes Bone Regeneration. ACS Appl Mater Interfaces.

[69] Zhang J, Liu X, Li H, Chen C, Hu B, Niu X (2016). Exosomes/tricalcium phosphate combination scaffolds can enhance bone regeneration by activating the PI3K/Akt signaling pathway. Stem Cell Res Ther.

[70] Vonk LA, van Dooremalen SFJ, Liv N, Klumperman J, Coffer PJ, Saris DBF (2018). Mesenchymal Stromal/stem Cell-derived Extracellular Vesicles Promote Human Cartilage Regeneration In Vitro. Theranostics.

[71] Gao Y, Liu S, Huang J, Guo W, Chen J, Zhang L (2014). The ECM-Cell Interaction of Cartilage Extracellular Matrix on Chondrocytes. Biomed Res Int.

[72] Mao G, Zhang Z, Hu S, Zhang Z, Chang Z, Huang Z (2018). Exosomes derived from miR-92a-3p-overexpressing human mesenchymal stem cells enhance chondrogenesis and suppress cartilage degradation via targeting WNT5A. Stem Cell Res Ther.

[73] Hosseini-Farahabadi S, Geetha-Loganathan P, Fu K, Nimmagadda S, Yang HJ, Richman JM (2013). Dual functions for WNT5A during cartilage development and in disease. Matrix Biol.

[74] Wang R, Xu B, Xu H (2018). TGF-β1 promoted chondrocyte proliferation by regulating Sp1 through MSC-exosomes derived miR-135b. Cell Cycle.

[75] Zhang S, Chu WC, Lai RC, Lim SK, Hui JHP, Toh WS (2016). Exosomes derived from human embryonic mesenchymal stem cells promote osteochondral regeneration. Osteoarthritis Cartilage.

[76] Zhang S, Chuah SJ, Lai RC, Hui JHP, Lim SK, Toh WS (2018). MSC exosomes mediate cartilage repair by enhancing proliferation, attenuating apoptosis and modulating immune reactivity. Biomaterials.

[77] Zhang S, Teo KYW, Chuah SJ, Lai RC, Lim SK, Toh WS (2019). MSC exosomes alleviate temporomandibular joint osteoarthritis by attenuating inflammation and restoring matrix homeostasis. Biomaterials.

[78] Wang Y, Yu D, Liu Z, Zhou F, Dai J, Wu B (2017). Exosomes from embryonic mesenchymal stem cells alleviate osteoarthritis through balancing synthesis and degradation of cartilage extracellular matrix. Stem Cell Res Ther.

[79] Yan L, Wu X (2020). Exosomes produced from 3D cultures of umbilical cord mesenchymal stem cells in a hollow-fiber bioreactor show improved osteochondral regeneration activity. Cell Biol Toxicol.

[80] Liu X, Yang Y, Li Y, Niu X, Zhao B, Wang Y (2017). Integration of stem cell-derived exosomes with in situ hydrogel glue as a promising tissue patch for articular cartilage regeneration. Nanoscale.

[81] Bishop ES, Mostafa S, Pakvasa M, Luu HH, Lee MJ, Wolf JM (2017). 3-D bioprinting technologies in tissue engineering and regenerative medicine: Current and future trends. Genes Dis.

[82] Chen P, Zheng L, Wang Y, Tao M, Xie Z, Xia C (2019). Desktop-stereolithography 3D printing of a radially oriented extracellular matrix/mesenchymal stem cell exosome bioink for osteochondral defect regeneration. Theranostics.

[83] Bruno S, Grange C, Deregibus MC, Calogero RA, Saviozzi S, Collino F (2009). Mesenchymal Stem Cell-Derived Microvesicles Protect Against Acute Tubular Injury. J Am Soc Nephrol.

[84] Zhao L, Hu C, Zhang P, Jiang H, Chen J (2019). Genetic communication by extracellular vesicles is an important mechanism underlying stem cell-based therapy-mediated protection against acute kidney injury. Stem Cell Res Ther.

[85] Wang S, Hong Q, Zhang C, Yang Y, Cai G, Chen X (2019). miRNAs in stem cell-derived extracellular vesicles for acute kidney injury treatment: comprehensive review of preclinical studies. Stem Cell Res Ther.

[86] Bruno S, Grange C, Collino F, Deregibus MC, Cantaluppi V, Biancone L (2012). Microvesicles Derived from Mesenchymal Stem Cells Enhance Survival in a Lethal Model of Acute Kidney Injury. PLoS One.

[87] Zhou Y, Xu H, Xu W, Wang B, Wu H, Tao Y (2013). Exosomes released by human umbilical cord mesenchymal stem cells protect against cisplatin-induced renal oxidative stress and apoptosis in vivo and in vitro. Stem Cell Res Ther.

[88] Tan CY, Lai RC, Wong W, Dan YY, Lim S, Ho HK (2014). Mesenchymal stem cell-derived exosomes promote hepatic regeneration in drug-induced liver injury models. Stem Cell Res Ther.

[89] Nong K, Wang W, Niu X, Hu B, Ma C, Bai Y (2016). Hepatoprotective effect of exosomes from human-induced pluripotent stem cell-derived mesenchymal stromal cells against hepatic ischemia-reperfusion injury in rats. Cytotherapy.

[90] Du Y, Li D, Han C, Wu H, Xu L, Zhang M (2017). Exosomes from Human-Induced Pluripotent Stem Cell-Derived Mesenchymal Stromal Cells (hiPSC-MSCs) Protect Liver against Hepatic Ischemia/ Reperfusion Injury via Activating Sphingosine Kinase and Sphingosine-1-Phosphate Signaling Pathway. Cellular Physiology and Biochemistry.

[91] Yao J, Zheng J, Cai J, Zeng K, Zhou C, Zhang J (2019). Extracellular vesicles derived from human umbilical cord mesenchymal stem cells alleviate rat hepatic ischemia-reperfusion injury by suppressing oxidative stress and neutrophil inflammatory response. FASEB J.

[92] Mardpour S, Ghanian MH, Sadeghi-abandansari H, Mardpour S, Nazari A, Shekari F (2019). Hydrogel-Mediated Sustained Systemic Delivery of Mesenchymal Stem Cell-Derived Extracellular Vesicles Improves Hepatic Regeneration in Chronic Liver Failure. ACS Appl Mater Interfaces.

[93] Zhao R, Liang H, Clarke E, Jackson C, Xue M (2016). Inflammation in Chronic Wounds. International Journal of Molecular Sciences.

[94] Shabbir A, Cox A, Rodriguez-Menocal L, Salgado M, Van Badiavas E (2015). Mesenchymal Stem Cell Exosomes Induce Proliferation and Migration of Normal and Chronic Wound Fibroblasts, and Enhance Angiogenesis In Vitro. Stem Cells Dev.

[95] Zhang J, Guan J, Niu X, Hu G, Guo S, Li Q (2015). Exosomes released from human induced pluripotent stem cells-derived MSCs facilitate cutaneous wound healing by promoting collagen synthesis and angiogenesis. J Transl Med.

[96] Choi JS, Lee Cho W, Choi YJ, Kim JD, Park H, Kim SY (2019). Functional recovery in photo-damaged human dermal fibroblasts by human adipose-derived stem cell extracellular vesicles. J Extracell Vesicles.

[97] Pelizzo G, Avanzini MA, Icaro Cornaglia A, De Silvestri A, Mantelli M, Travaglino P (2018). Extracellular vesicles derived from mesenchymal cells: perspective treatment for cutaneous wound healing in pediatrics. Regen Med.

[98] Zhang B, Shi Y, Gong A, Pan Z, Shi H, Yang H (2016). HucMSC Exosome-Delivered 14-3-3ζ Orchestrates Self-Control of the Wnt Response via Modulation of YAP During Cutaneous Regeneration. Stem Cells.

[99] Zhang B, Wu X, Zhang X, Sun Y, Yan Y, Shi H (2015). Human Umbilical Cord Mesenchymal Stem Cell Exosomes Enhance Angiogenesis Through the Wnt4/β-Catenin Pathway. Stem Cells Transl Med.

[100] Fang S, Xu C, Zhang Y, Xue C, Yang C, Bi H (2016). Umbilical Cord-Derived Mesenchymal Stem Cell-Derived Exosomal MicroRNAs Suppress Myofibroblast Differentiation by Inhibiting the Transforming Growth Factor-β/SMAD2 Pathway During Wound Healing. Stem Cells Transl Med.

[101] Pi Y, Xia B, Jin M, Jin W, Lou G (2021). Exosomes: Powerful weapon for cancer nano-immunoengineering. Biochem Pharmacol.

[102] Zhang F, Guo J, Zhang Z, Qian Y, Wang G, Duan M (2022). Mesenchymal stem cell-derived exosome: A tumor regulator and carrier for targeted tumor therapy. Cancer Lett.

[103] Pakravan K, Babashah S, Sadeghizadeh M, Mowla SJ, Mossahebi-Mohammadi M, Ataei F (2017). MicroRNA-100 shuttled by mesenchymal stem cell-derived exosomes suppresses in vitro angiogenesis through modulating the mTOR/HIF-1α/VEGF signaling axis in breast cancer cells. Cell Oncol (Dordr).

[104] Katakowski M, Buller B, Zheng X, Lu Y, Rogers T, Osobamiro O (2013). Exosomes from marrow stromal cells expressing miR-146b inhibit glioma growth. Cancer Lett.

[105] Yao X, Mao Y, Wu D, Zhu Y, Lu J, Huang Y (2021). Exosomal circ_0030167 derived from BM-MSCs inhibits the invasion, migration, proliferation and stemness of pancreatic cancer cells by sponging miR-338-5p and targeting the Wif1/Wnt8/β-catenin axis. Cancer Lett.

[106] Shao J, Zaro J, Shen Y (2020). Advances in Exosome-Based Drug Delivery and Tumor Targeting: From Tissue Distribution to Intracellular Fate. Int J Nanomedicine.

[107] Alvarez-Erviti L, Seow Y, Yin H, Betts C, Lakhal S, Wood MJA (2011). Delivery of siRNA to the mouse brain by systemic injection of targeted exosomes. Nat Biotechnol.

[108] Kalluri R, LeBleu VS (2020). The biology, function, and biomedical applications of exosomes. Science.

[109] Tian Y, Li S, Song J, Ji T, Zhu M, Anderson GJ (2014). A doxorubicin delivery platform using engineered natural membrane vesicle exosomes for targeted tumor therapy. Biomaterials.

[110] Wang J, Li G, Tu C, Chen X, Yang B, Huo Y (2020). High-throughput single-cell analysis of exosome mediated dual drug delivery,in vivo fate and synergistic tumor therapy. Nanoscale.

[111] Harrell CR, Jovicic N, Djonov V, Arsenijevic N, Volarevic V (2019). Mesenchymal Stem Cell-Derived Exosomes and Other Extracellular Vesicles as New Remedies in the Therapy of Inflammatory Diseases. Cells.

[112] Yeo RWY, Lai RC, Zhang B, Tan SS, Yin Y, Teh BJ (2013). Mesenchymal stem cell: An efficient mass producer of exosomes for drug delivery. Adv Drug Deliv Rev.

[113] Yang N, Ding Y, Zhang Y, Wang B, Zhao X, Cheng K (2018). Surface Functionalization of Polymeric Nanoparticles with Umbilical Cord-Derived Mesenchymal Stem Cell Membrane for Tumor-Targeted Therapy. ACS Appl Mater Interfaces.

[114] El-Haibi CP, Karnoub AE (2010). Mesenchymal Stem Cells in the Pathogenesis and Therapy of Breast Cancer. J Mammary Gland Biol Neoplasia.

[115] Walker S, Busatto S, Pham A, Tian M, Suh A, Carson K (2019). Extracellular vesicle-based drug delivery systems for cancer treatment. Theranostics.

[116] Jain RK, Stylianopoulos T (2010). Delivering nanomedicine to solid tumors. Nat Rev Clin Oncol.

[117] Ankrum JA, Ong JF, Karp JM (2014). Mesenchymal stem cells: immune evasive, not immune privileged. Nat Biotechnol.

[118] Wei H, Chen J, Wang S, Fu F, Zhu X, Wu C (2019). A Nanodrug Consisting Of Doxorubicin And Exosome Derived From Mesenchymal Stem Cells For Osteosarcoma Treatment In Vitro. Int J Nanomedicine.

[119] Rosenkrans Z, Thickens A, Kink J, Eduardo A, Hematti E, Hernandez R (2024). Investigating the In Vivo Biodistribution of Extracellular Vesicles Isolated from Various Human Cell Sources Using Positron Emission Tomography. Mol. Pharmaceutics.

[120] Zhang S, Guo W (2021). β-Elemene Enhances the Sensitivity of Osteosarcoma Cells to Doxorubicin via Downregulation of Peroxiredoxin-1. Onco Targets Ther.

[121] Bagheri E, Abnous K, Farzad SA, Taghdisi SM, Ramezani M, Alibolandi M (2020). Targeted doxorubicin-loaded mesenchymal stem cells-derived exosomes as a versatile platform for fighting against colorectal cancer. Life Sci.

[122] Song JH, Kim J, Lee MN, Oh S, Piao X, Wang Z (2022). Isolation of High Purity Mouse Mesenchymal Stem Cells through Depleting Macrophages Using Liposomal Clodronate. Tissue Eng Regen Med.

[123] Melzer C, Rehn V, Yang Y, Bähre H, von der Ohe J, Hass R (2019). Taxol-Loaded MSC-Derived Exosomes Provide a Therapeutic Vehicle to Target Metastatic Breast Cancer and Other Carcinoma Cells. Cancers (Basel).

[124] Zhou Y, Zhou W, Chen X, Wang Q, Li C, Chen Q (2020). Bone marrow mesenchymal stem cells-derived exosomes for penetrating and targeted chemotherapy of pancreatic cancer. Acta Pharm Sin B.

[125] Rauf A, Patel S, Imran M, Maalik A, Arshad MU, Saeed F (2018). Honokiol: An anticancer lignan. Biomed Pharmacother.

[126] Zhou J, Ren Y, Tan L, Song X, Wang M, Li Y (2020). Norcantharidin: research advances in pharmaceutical activities and derivatives in recent years. Biomed Pharmacother.

[127] Liang L, Zhao L, Wang Y, Wang Y (2021). Treatment for Hepatocellular Carcinoma Is Enhanced When Norcantharidin Is Encapsulated in Exosomes Derived from Bone Marrow Mesenchymal Stem Cells. Mol Pharm.

[128] Lou G, Chen L, Xia C, Wang W, Qi J, Li A (2020). MiR-199a-modified exosomes from adipose tissue-derived mesenchymal stem cells improve hepatocellular carcinoma chemosensitivity through mTOR pathway. J Exp Clin Cancer Res.

[129] Yu L, Gui S, Liu Y, Qiu X, Zhang G, Zhang X (2019). Exosomes derived from microRNA-199a-overexpressing mesenchymal stem cells inhibit glioma progression by down-regulating AGAP2. Aging (Albany NY).

[130] Munoz JL, Bliss SA, Greco SJ, Ramkissoon SH, Ligon KL, Rameshwar P (2013). Delivery of Functional Anti-miR-9 by Mesenchymal Stem Cell-derived Exosomes to Glioblastoma Multiforme Cells Conferred Chemosensitivity. Mol Ther Nucleic Acids.

[131] Li H, Yang C, Shi Y, Zhao L (2018). Exosomes derived from siRNA against GRP78 modified bone-marrow-derived mesenchymal stem cells suppress Sorafenib resistance in hepatocellular carcinoma. J Nanobiotechnology.

